# *In Silico* design and evaluation of a multi-epitope vaccine candidate against *Toxoplasma gondii* for humans

**DOI:** 10.3389/fcimb.2026.1905185

**Published:** 2026-08-17

**Authors:** Chenchen Yi, Yu Shen, Ye Luo, Qin Wang, Xiaomei Tong

**Affiliations:** 1 Assisted Reproduction Unit, Department of Obstetrics and Gynecology, Sir Run Run Shaw Hospital, School of Medicine, Zhejiang University, Hangzhou, China; 2 Hangzhou Center for Disease Control and Prevention (Hangzhou Health Supervision Institution), Hangzhou, China; 3 Zhejiang Key Laboratory of High-level Biosafety and Biomedical Transformation, Hangzhou, China; 4 Zhejiang Key Laboratory of Precise Protection and Promotion of Fertility, Hangzhou, China; 5 Zhejiang Provincial Clinical Research Center for Reproductive Health and Disease, Hangzhou, China

**Keywords:** immune response, immunoinformatics, multi-epitope vaccine, *Toxoplasma gondii*, zoonotic diseases

## Abstract

**Introduction:**

*Toxoplasma gondii* is a significant zoonotic pathogen responsible for severe disease in immunocompromised individuals, adverse pregnancy outcomes, and substantial economic losses in the livestock industry. Given the limitations of current therapeutic strategies and vaccines, this study utilized immunoinformatics and reverse vaccinology approaches to design a multi-epitope candidate vaccine.

**Methods:**

A total of 49 T. gondii proteins from the SAG, GRA, MIC, and ROP families were screened. Epitopes were selected based on antigenicity, immunogenicity, cytokine-inducing potential, toxicity, and allergenicity. The selected epitopes were assembled using AAY, GPGPG, and KK linkers, with the 50S ribosomal protein L7/L12 as an adjuvant. Physicochemical properties, molecular docking, molecular dynamics simulations, immune simulations, and codon optimization were subsequently evaluated.

**Results:**

We identified 9 cytotoxic T lymphocyte (CTL), 6 helper T lymphocyte (HTL), and 11 B-cell epitopes. The finalized 556-amino-acid construct (61.13 kDa) demonstrated favorable antigenicity (VaxiJen score: 0.6843), stability (instability index: 39.05), solubility (0.663), and hydrophilicity (GRAVY: -0.502), while maintaining safety profiles. Molecular docking and dynamics simulations confirmed robust and stable binding to TLR2 (-32.8 kcal/mol) and TLR4 (-72.37 kcal/mol). Furthermore, immune simulations predicted a strong, memory-forming humoral and cellular immune response. Codon optimization (CAI: 0.93, GC content: 56.43%) suggested high expression efficiency in *Escherichia coli*.

**Discussion:**

The rationally designed multi-epitope vaccine demonstrates robust theoretical potential to elicit comprehensive, long-lasting immunity in humans, although its safety and effectiveness require additional experimental validation.

## 1 Introduction

*Toxoplasma gondii* (*T. gondii*) is an obligate intracellular parasite capable of infecting nearly all warm-blooded animals worldwide, causing zoonotic toxoplasmosis (Dubey, 2009). Approximately 25–50% of the global population is estimated to be infected with *T. gondii* (Flegr et al., 2014). Most infected individuals remain asymptomatic or exhibit mild, non-specific symptoms. However, those with compromised immune systems, such as organ transplant recipients, cancer patients, and individuals with AIDS may develop severe manifestations, and infection in pregnant women may lead to serious adverse pregnancy outcomes (Kur et al., 2009; Robert-Gangneux and Dardé, 2012). Besides affecting human health, toxoplasmosis also causes significant economic losses in the livestock industry, especially in the breeding of small ruminants and pigs, primarily due to reproductive disorders like miscarriage and stillbirth (Foroutan et al., 2019; Dubey et al., 2005; Nayeri et al., 2021).

To address the threat of *T. gondii* to human health, researchers have proposed various drug treatment regimens. Among these, the combination of pyrimethamine and sulfadiazine (pyr-sulf) is currently regarded as the gold standard for the treatment of toxoplasmosis. Additionally, clindamycin, atovaquone, clarithromycin, spiramycin, and azithromycin have also been reported to have anti- *T. gondii* activity (Alday and Doggett, 2017; Dubey, 2008). However, the existing therapies present notable drawbacks, including prolonged treatment duration, significant side effects, and effectiveness only for acute infections (Dunay et al., 2018). Consequently, in the absence of ideal therapeutic drugs, developing more effective and safer prevention strategies for toxoplasmosis has become an urgent priority.

In recent years, various types of T. gondii vaccines have been developed, including attenuated live vaccines (Wang et al., 2024), inactivated vaccines (Zorgi et al., 2011), subunit vaccines (Tian et al., 2022), and DNA vaccines (Yu et al., 2022). Attenuated live vaccines such as the S48 strain (Toxovax^®^) have shown protective efficacy in sheep, but their safety issues have limited their wider application (Buxton and Innes, 1995), while inactivated vaccines have been proven ineffective in most infection models (Saavedra et al., 2004; Kur et al., 2009). Subunit and DNA vaccines are the categories that have received more extensive research attention in recent years (Zhang et al., 2022). For instance, recombinant GRA7 and GRA12 proteins formulated with PLGA nanoparticles (Sun et al., 2023), as well as DNA vaccines encoding SAG1 and ROP18 (Fang et al., 2011; Zhu et al., 2020), have induced Th1-biased immune responses and prolonged survival in mice, though complete protection has rarely been achieved. Multi-epitope designs, such as the ROM4 epitope-based vaccine, have elicited high levels of IFN-γ responses yet conferred only partial protection against challenge (Han et al., 2017). Despite these efforts, no subunit or multi-epitope candidates have entered human clinical trials, and Toxovax^®^ remains the only licensed vaccine, with its use limited to veterinary purposes for sheep. The respective limitations of the aforementioned vaccines indicate that an ideal *T. gondii* vaccine should exhibit safety, broad-spectrum efficacy and high efficiency. In recent years, bioinformatics-driven multi-epitope vaccine design has represented an important innovation in the field of vaccine development, primarily due to its core advantage of integrating multiple antigen epitopes through computational biology methods to address the limitations of traditional vaccines (Soto et al., 2022; Lou et al., 2022; Nosrati et al., 2019). The multi-epitope vaccine strategy based on immunoinformatics can simultaneously activate CD8+ T cells, CD4+ T cells and B cell-mediated immune responses, while posing no risk of virulence rebound. Currently, this strategy has been applied in the vaccine development for various pathogens, including HIV (Pandey et al., 2018), H7N9 influenza virus (Tarrahimofrad et al., 2021) and Brucella (Wang et al., 2023).

In this study, we designed a human-targeted multi-epitope candidate vaccine against *T. gondii* by integrating reverse vaccinology with immunoinformatics techniques. Initially, from 49 types of proteins derived from *T. gondii*, the ideal T-cell (CTL and HTL) and B-cell epitopes suitable for the construction of the final vaccine were selected. On this basis, the binding mode of the designed vaccine with immune receptors, the characteristics of the immune response, and the potential for large-scale production were systematically evaluated. In conclusion, this study not only provides a theoretical basis and data support for the subsequent clinical T. gondii vaccine research, but also offers a scalable strategy for designing vaccines against other similar zoonotic pathogens threatening human health.

## 2 Materials and methods

### 2.1 Protein sequences retrieval

In this study, a total of 49 widely studied T. gondii antigens were selected as the initial dataset for this research. These antigens belong to the main functional families, including surface antigens (SAGs), dense granule proteins (GRAs), microneme proteins (MICs), and rhoptry proteins (ROPs). The complete amino acids sequences of all T. gondii proteins were obtained from the ToxoDB database (https://toxodb.org/toxo/app) and the NCBI (National Center for Biotechnology Information) database (https://www.ncbi.nlm.nih.gov/). The corresponding accession numbers and FASTA sequences are provided in Sheet 1 of the **Supplementary Materials**. All 49 proteins were directly used for the prediction of T cell and B cell epitopes.

### 2.2 Prediction of cytotoxic T lymphocyte epitopes

The CTL epitopes (9-mer) were predicted and analyzed using the NetMHC pan4.1 server (Reynisson et al., 2020) (https://services.healthtech.dtu.dk/services/NetMHCpan-4.1/). The predicted epitopes are the default HLA representative subtypes (HLA-A*01:01,HLA-A*02:01,HLA-A*03:01,HLA-A*24:02,HLA-A*26:01,HLA-B*07:02,HLA-B*08:01,HLA-B*27:05,HLA-B*39:01,HLA-B*40:01,HLA-B*58:01,HLA-B*15:01) of the server and the selection criteria were set as epitopes with a percentage rank < 0.5 and a MHC binding affinity level < 50 nM. Subsequently, the immunogenicity of the predicted epitopes was evaluated through the IEDB I-type immunogenicity analysis server (Calis et al., 2013), and the epitopes with an immunogenicity score > 0 were retained. On this basis, the VaxiJen 2.0 server (https://www.ddg-pharmfac.net/vaxijen/VaxiJen/VaxiJen.html) was used to predict the antigenicity of the selected epitopes, with 0.5 set as the positive judgment threshold (Doytchinova and Flower, 2007). Then, the selected epitopes were subjected to toxicity assessment through the ToxinPred (Gupta et al., 2013) (https://webs.iiitd.edu.in/raghava/toxinpred/) server (using default parameters), as well as allergen characteristic analysis through AllerTOP v.2.1 (Dimitrov et al., 2014) (https://www.ddg-pharmfac.net/allertop_test/) and AlgPred 2.0 (Sharma et al., 2021) (https://webs.iiitd.edu.in/raghava/algpred2/batch.html) servers (both using default parameters).The epitopes determined through the above multiple screening process were finally identified as the CTL dominant immunogenic epitopes for constructing a multi-epitope vaccine.

### 2.3 Prediction of helper T lymphocytes epitopes

We used the NetMHCII pan4.3 server (Nilsson et al., 2023) (https://services.healthtech.dtu.dk/services/NetMHCIIpan-4.3/) to predict the HTL epitopes (15-mer) for these antigens and selected five of the most common HLA II class alleles as binding targets (HLA-DRB1_0101, HLA-DRB1_0401, HLA-DRB1_0701, HLA-DRB1_1101, HLA-DRB1_1501). Potential HTL epitopes were screened by percentage ranking, and epitopes with a percentage rank < 1 were selected for further study. Subsequently, the antigenicity of the epitopes was evaluated using the VaxiJen v2.0 server (with a threshold set at 0.5). To assess the potential of the selected epitopes to stimulate cytokine secretion, the IFNepitope (Dhanda et al., 2013b) (https://webs.iiitd.edu.in/raghava/ifnepitope/index.php), IL4Pred (Dhanda et al., 2013a) (https://webs.iiitd.edu.in/raghava/il4pred/), and IL-10Pred (Nagpal et al., 2017) (https://webs.iiitd.edu.in/raghava/il10pred/) online tools (with default parameters maintained) were used to evaluate their ability to induce IFN-γ, IL-4, and IL-10 production. Then, the selected epitopes were subjected to toxicity assessment through the ToxinPred server (using default parameters), as well as allergen characteristic analysis through AllerTOP v.2.1 and AlgPred 2.0 servers (both using default parameters). Finally, the multi-epitope combinations determined by the above screening criteria were classified as the dominant immunogenic HTL epitopes.

### 2.4 Prediction of B-cell epitopes

The ABCpred server (https://webs.iiitd.edu.in/raghava/abcpred/) was used for the prediction analysis of linear B-cell epitopes (Saha and Raghava, 2006). This server is based on an artificial neural network algorithm and has a prediction accuracy rate of 65.93%. In the experiment, the epitope length was set to 16 amino acid residues, the screening threshold was 0.5, but only the candidate epitopes with a prediction score exceeding 0.9 were selected. Then, the antigenicity of these epitopes was evaluated using the VaxiJen v2.0 server (with a threshold of 0.5). Subsequently, the selected epitopes were subjected to toxicity assessment through the ToxinPred server (using default parameters), as well as allergen characteristic analysis through AllerTOP v.2.1 and AlgPred 2.0 servers (both using default parameters). Finally, the non-toxic and non-allergenic B-cell epitopes were selected as the advantageous B-cell epitopes for constructing a multi-epitope vaccine.

### 2.5 Vaccine construction

The selected immunodominant CTL, HTL and B cell epitopes were fused through the AAY, GPGPG and KK linker segments to construct a multi-epitope subunit vaccine (Dong et al., 2020; Mahapatra et al., 2020; Pandey et al., 2018). To enhance immunogenicity, the adjuvant 50S ribosomal protein L7/L12 (NCBI ID: P9WHE3) was linked to the N-terminal of the vaccine using the EAAAK linker segment (Mahapatra et al., 2020).

### 2.6 Homology analysis

To ensure the safety of the vaccine candidate and minimize the risk of cross-reactivity or autoimmunity in the host, a homology analysis was performed. The full-length amino acid sequence of the multi-epitope vaccine was aligned against the human proteome (Homo sapiens, taxid: 9606) using the online BLASTp tool via the NCBI server (https://blast.ncbi.nlm.nih.gov/Blast.cgi). The Reference proteins (refseq_protein) database was selected as the alignment target with default parameters.

### 2.7 Features of vaccine construct

The antigenicity of the candidate vaccine was predicted using the VaxiJen 2.0 server, with a threshold set at 0.5. Its allergenicity was evaluated through the AllerTOP v.2.1 and AlgPred 2.0 servers, and its toxicity was assessed through the ToxinPred2 (Sharma et al., 2022) (https://webs.iiitd.edu.in/raghava/toxinpred2/algo.html) server. Subsequently, the physicochemical properties of the vaccine, including molecular weight, instability index, amino acid composition, theoretical isoelectric point (PI), aliphatic index, grand average of hydropathicity and half-life, were analyzed using the ProtParam tool (Wilkins et al., 1999) (https://web.expasy.org/protparam/). The potential for soluble expression of the candidate vaccine was evaluated through the Protein-Sol server (Hebditch et al., 2017) (https://protein-sol.manchester.ac.uk/). Additionally, the possible transmembrane helices and signal peptides in the vaccine were predicted using the DeepTMHMM (https://services.healthtech.dtu.dk/services/DeepTMHMM-1.0/) and SignalP6.0 (https://services.healthtech.dtu.dk/services/SignalP-6.0/) servers respectively.

### 2.8 Secondary and tertiary structure predictions, tertiary structure refinement and validation

The secondary structure of the multi-epitope vaccine was predicted using the PSIPRED server (Buchan and Jones, 2019) (https://bioinf.cs.ucl.ac.uk/psipred/). Subsequently, its three-dimensional structure was determined using the AlphaFold Server (Abramson et al., 2024) (https://alphafoldserver.com/). The three-dimensional model was optimized using the GalaxyRefine server (Heo et al., 2016; Mirela-Bota et al., 2020) (https://galaxy.seoklab.org/cgi-bin/submit.cgi?type=REFINE). Additionally, the Ramachandran plot was generated using the PROCHECK module of the SAVES v6.1 server (https://saves.mbi.ucla.edu/), and the quality of the optimized model was comprehensively verified by combining the ERRAT score and the Z score from the ProSA-web server (Wiederstein and Sippl, 2007) (https://prosa.services.came.sbg.ac.at/prosa.php).

### 2.9 Molecular docking and dynamic simulation

The binding affinity between the vaccine and the immune receptors was evaluated using molecular docking technology. Firstly, the crystal structure files of TLR2 (PDB ID: 6NIG) and TLR4 (PDB ID: 4G8A) were obtained from the RCSB PDB database (https://www.rcsb.org/). Subsequently, through the HawkDock server (Weng et al., 2019) (https://cadd.zju.edu.cn/hawkdock/) platform, the molecular interaction mechanism of the multi-epitope vaccine with TLR2 and TLR4 was systematically studied. Moreover, the PDBsum server (https://www.ebi.ac.uk/thornton-srv/databases/cgi-bin/pdbsum/GetPage.pl?pdbcode=index.html) was used to conduct in-depth analysis of the key interacting residues in the docking complex (Laskowski et al., 2017). Finally, molecular dynamics (MD) simulations were conducted using the iMODS network server (López-Blanco et al., 2014) (https://imods.iqf.csic.es/) to assess the stability of the vaccine-receptor complex.

### 2.10 Immune simulation

We conducted a computerized immune simulation on the C-ImmSim server (https://kraken.iac.rm.cnr.it/C-IMMSIM/index.php) to evaluate the immune response of multi-epitope vaccines (Rapin et al., 2010). The dosing regimen used in this study was 1000 vaccine units per dose, administered once every 4 weeks, for a total of 3 doses. The total simulation steps were set at 1050 steps, with each step representing 8 hours. The injections were carried out at time steps 1, 84, and 168.

### 2.11 Population coverage analysis

To evaluate the potential global efficacy and applicability of the designed vaccine across diverse human populations, the population coverage of the selected CTL and HTL epitopes along with their respective HLA binding alleles was calculated using the Population Coverage tool (https://tools.iedb.org/population/) provided by the IEDB database.

### 2.12 Codon optimization and cloning

The target sequence was reverse translated using the online tool VectorBuilder (https://www.vectorbuilder.cn/tool/codon-optimization.html) and codon optimization was performed in the *Escherichia coli* K12 host strain. To evaluate the transcription and translation efficiency, we analyzed the GC content percentage and codon adaptation index (CAI). Additionally, restriction enzyme sites of BamHI and XhoI were inserted at the N-terminal and C-terminal of the cDNA using the SnapGene software, so that it could be cloned into the pET-28a (+) vector. This in silico cloning strategy was designed to support the development of the candidate construct as a recombinant protein vaccine platform.

## 3 Results

### 3.1 Screening and construction of multi-epitope vaccine dominant epitopes

A total of 49 T. gondii proteins were subjected to epitope prediction. Through successive screening and cascade evaluation of antigenicity, immunogenicity, toxicity, allergenicity, and the ability to induce cytokines IFN-γ, IL-4, and IL-10, 9 CTL epitopes (**Table 1**), 6 HTL epitopes (**Table 2**), and 11 linear B-cell epitopes (**Table 3**) were finally obtained, successfully meeting all strict criteria. These 26 identified epitopes were systematically used as building blocks for the multi-epitope vaccine construct. Furthermore, the results of BLASTp (**Supplementary Material Table 2**) indicate that the final constructed vaccine has no homology with human proteins.

**Table 1:** Screening of CTL epitopes.

Protein	Start	Peptide	Alleles	%Rank_EL	Aff (nM) bindlevel	Immunogenicity	Antigenicity	Toxicity	Allergenicity
SAG2C	43	LLVILTASF	HLA-B*15:01	0.45	15.18	0.10942	0.7333	Non-Toxin	Non-Allergen
GRA14	232	IMEDHVASV	HLA-A*02:01	0.038	11.45	0.06154	1.2279	Non-Toxin	Non-Allergen
GRA17	116	IMWELRHFL	HLA-A*02:01	0.059	4.08	0.30627	0.7064	Non-Toxin	Non-Allergen
MIC1	125	ATRHEILSK	HLA-A*03:01	0.027	37.54	0.16611	1.1542	Non-Toxin	Non-Allergen
MIC2	96	KQFLHTFLM	HLA-B*15:01	0.26	30.83	0.1872	1.1837	Non-Toxin	Non-Allergen
MIC11	5	KLSVVSITL	HLA-A*02:01	0.122	26.71	0.00731	1.5976	Non-Toxin	Non-Allergen
ROP21	486	KLPNYFISI	HLA-A*02:01	0.07	6.72	0.11215	0.9105	Non-Toxin	Non-Allergen
	323	RSTRTNEKF	HLA-B*58:01	0.097	22.99	0.05489	1.1592	Non-Toxin	Non-Allergen
NTPase- II	613	NHFKWNVTI	HLA-B*39:01	0.012	14.99	0.08813	0.6239	Non-Toxin	Non-Allergen

**Table 2:** Screening of HTL epitopes.

Protein	Start	Peptide	Alleles	%Rank_EL	Aff (nM) bindlevel	Antigenicity	Toxicity	Allergenicity	IFN-γ	IL-4	IL-10
GRA14	301	MALLKYGTPRVRAFL	DRB1_1501	0.45	6.69	0.6828	Non-Toxin	Non-Allergen	+	+	+
GRA17	208	EAHIEVMKGRTSNMV	DRB1_0101	0.66	6.72	0.6032	Non-Toxin	Non-Allergen	+	+	+
ROP22	294	ELRIWRHLPSSRAEQ	DRB1_0101	0.39	3.74	0.5001	Non-Toxin	Non-Allergen	+	+	+
	293	WELRIWRHLPSSRAE	DRB1_0101	0.97	4.47	1.1273	Non-Toxin	Non-Allergen	+	+	+
CDPK1	286	RKMLTYVPSMRISAR	DRB1_0701	0.09	3.81	0.6555	Non-Toxin	Non-Allergen	+	+	+
HSP60	238	EKPFVLLYDKRISSV	DRB1_0101	0.33	12.81	1.0814	Non-Toxin	Non-Allergen	+	+	+

+ Indicates that it can induce the production of interleukins or cytokines. IFN-γ inducer, IL-4 inducer, IL-10 inducer.

**Table 3:** Screening of B-cell epitopes.

Protein	Start	Peptide	Score	Antigenicity	Toxicity	Allergenicity
SAG2B	94	SKSEEGSKAMRSTKSE	0.9	1.2339	Non-Toxin	Non-Allergen
GRA8	197	PSDISTHVRGAIRRQP	0.93	1.0588	Non-Toxin	Non-Allergen
GRA16	174	QSLAMRPRTMGQTMKS	0.94	1.032	Non-Toxin	Non-Allergen
GRA17	117	MWELRHFLYGTAKVNP	0.94	0.5691	Non-Toxin	Non-Allergen
MIC6	231	ESDSTEPGRDQERRTP	0.9	1.5819	Non-Toxin	Non-Allergen
ROP7	361	PAIEIDMGRFVEELYE	0.91	0.926	Non-Toxin	Non-Allergen
ROP16	97	GRTGGSAGAREERRSP	0.94	1.5778	Non-Toxin	Non-Allergen
ROP54	328	TDTPGTGPAHDARVSL	0.95	1.2624	Non-Toxin	Non-Allergen
MYR1	562	ASRTDAPVEAGQGDVR	0.91	0.7291	Non-Toxin	Non-Allergen
PLP1	264	NQTRITHPKSKAQHQK	0.91	1.2973	Non-Toxin	Non-Allergen
Toxofilin	30	LSEGMKRLSMRGRSPS	0.91	0.6571	Non-Toxin	Non-Allergen

### 3.2 Vaccine construct and features

**Figure 1:**
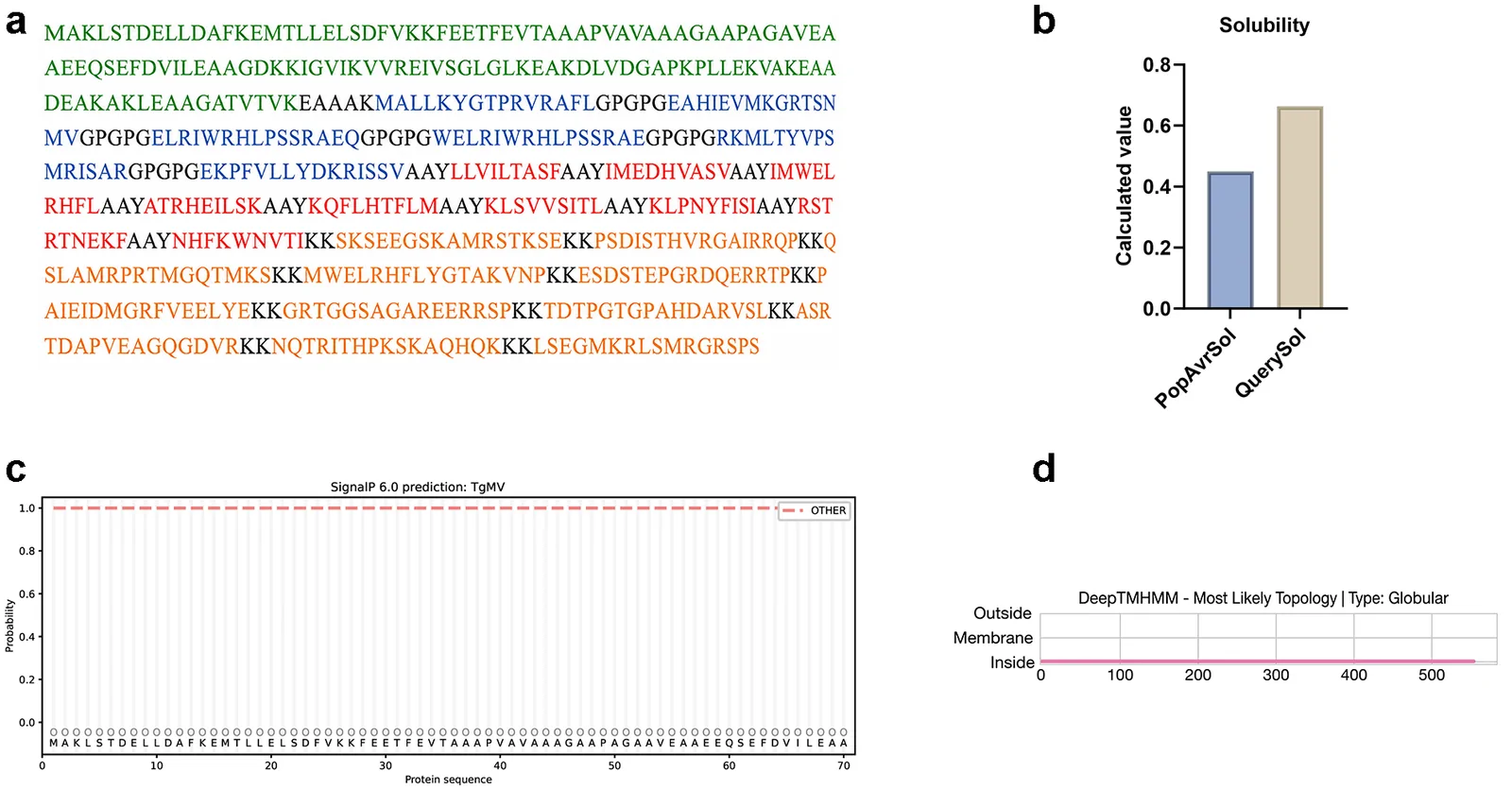
Sequence composition, solubility, signal peptide and transmembrane structure analysis of multi-epitope vaccine. **(A)** Amino acid sequence. Green represents the adjuvant, blue represents the HTL epitope, red represents the CTL epitope, orange represents the B-cell epitope, and black represents the linker. **(B)** The solubility predicted by the Protein-Sol server. **(C)** The signal peptide predicted by the SignalP6.0 server. **(D)** The transmembrane structure predicted by the DeepTMHMM server.

The amino acid sequence of the final multi-epitope vaccine is shown in **Figure 1A**. According to the prediction results of VaxiJen 2.0, the antigenicity score of this vaccine is 0.6843, indicating its potential antigenicity. The results from AllerTOP v.2.1, AlgPred 2.0 and ToxinPred2 servers show that this vaccine is non-allergenic and non-toxic. The physicochemical analysis indicates that the vaccine consists of 556 amino acids, with a molecular weight of 61.13 kDa and a theoretical isoelectric point of 9.96. The vaccine’s instability index is 39.05, which is below the threshold of 40, indicating its stable nature. The vaccine’s aliphatic index is 73.65, suggesting its thermal stability. The vaccine’s GRAVY value is -0.502 (< 0), indicating its hydrophilicity. The estimated half-life of the vaccine in mammalian reticulocytes is 30 hours, in yeast it exceeds 20 hours, and in *Escherichia coli* it exceeds 10 hours. According to the prediction results of Protein-Sol server, the vaccine has excellent solubility of 0.663 (**Figure 1B**), higher than the threshold of 0.45. Moreover, no signal peptide (**Figure 1C**) and transmembrane helix (**Figure 1D**) were detected in the vaccine construct, suggesting that there is no obvious heterologous expression barrier in the host expression system, which is conducive to subsequent recombinant expression and purification.

### 3.3 Secondary and tertiary structure prediction, tertiary structure optimization and verification

**Figure 2:**
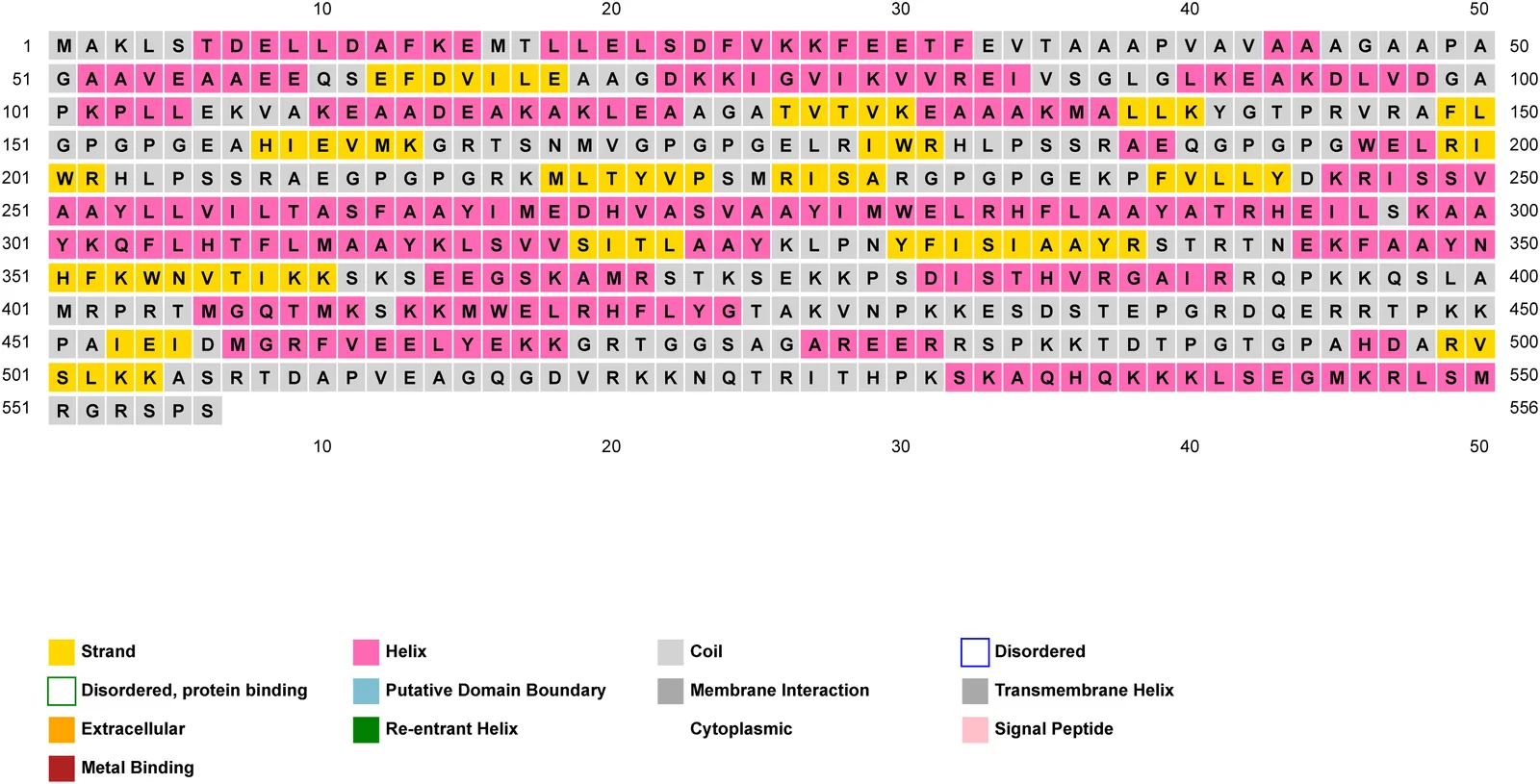
Use the PSIPRED tool to predict the secondary structure of multi-epitope vaccine.

**Figure 3:**
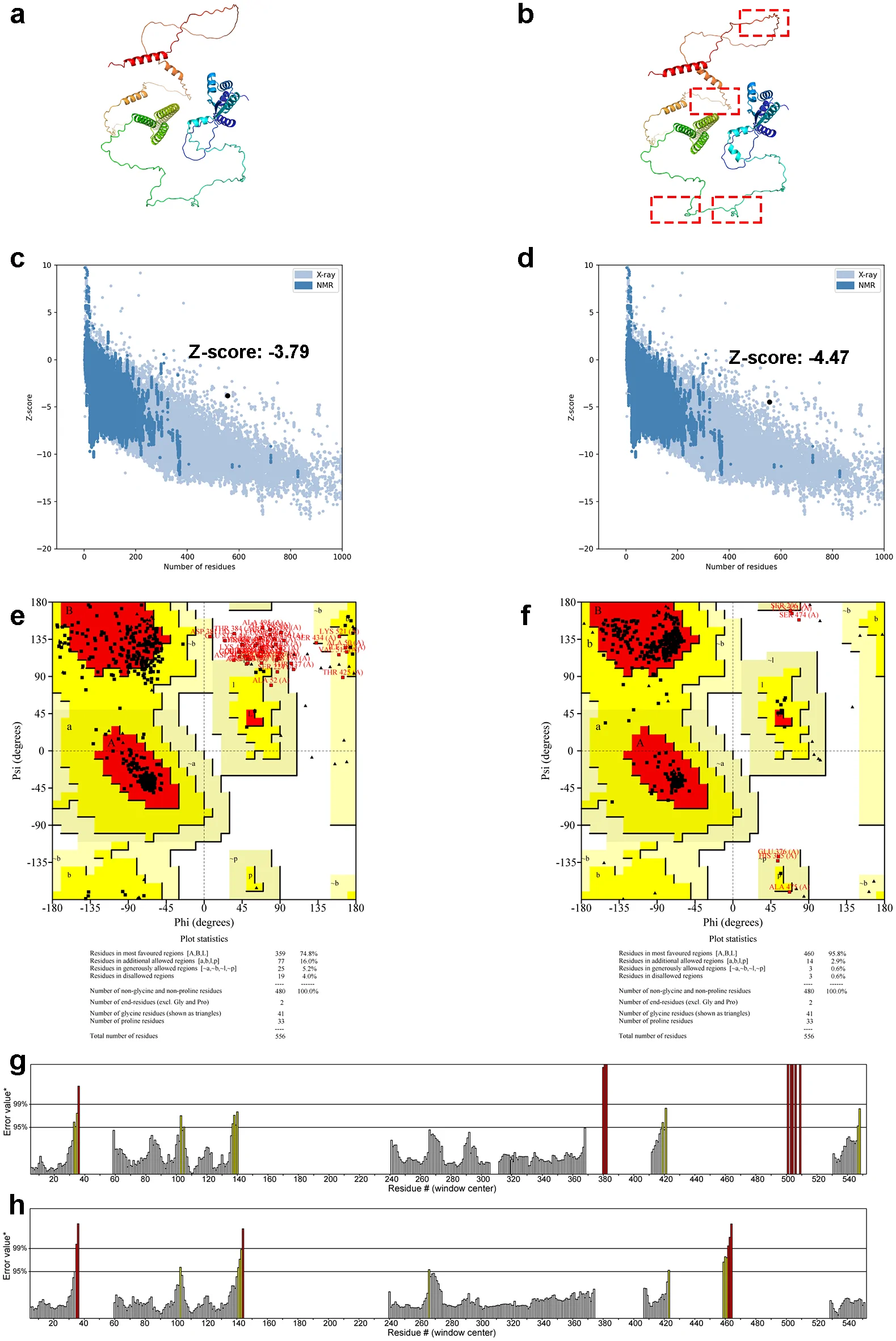
Quality assessment of the tertiary structure of multi-epitope vaccine. **(A, B)** The initial and optimized 3D models of multi-epitope vaccines. **(C, D)** The initial and optimized z-score plots of the multi-epitope vaccine predicted by the ProSA-web server. **(E, F)** The Ramachandran plots of the initial and optimized models of multi-epitope vaccines generated by the PROCHECK server. **(G, H)** The ERRAT scores of the initial and optimized models of multi-epitope vaccines generated by the ERRAT server.

Through analysis using the PSIPRED4.0 server, the secondary structure of this vaccine consists of 43.88% α-helix, 13.85% β-sheet, and 42.27% random coil (**Figure 2**). We generated the initial 3D structure of the vaccine using the AlphaFold online server (**Figure 3A**) and optimized it through the GalaxyRefine server (**Figure 3B**). The GDTHA value of the best optimized vaccine model was 0.8629, the RMSD value was 0.722, the MolProbity value was 0.979, and the Clash score was 1.0. Subsequently, we used the PROCHECK, ERRAT, and ProSA-web servers to evaluate the quality of the vaccine model and verify potential errors. The PROCHECK analysis showed that in the Ramachandran plot of the initial vaccine, 74.8% of the residues were in the most favorable region, 16% in the additional allowed region, 5.2% in the lenient allowed region, and 4% in the disallowed region (**Figure 3E**). The ERRAT score was 92.0863 (**Figure 3G**), and the Z score calculated by the ProSA-web server was -3.79 (**Figure 3C**). After optimization, the quality of the vaccine structure significantly improved: 95.8% of the residues were in the most favorable region, 2.9% in the additional allowed region, 0.6% in the lenient allowed region, and only 0.6% in the disallowed region (**Figure 3F**). The ERRAT score increased to 94.9664 (**Figure 3H**), and the Z score of ProSA-web improved to -4.47 (**Figure 3D**).

### 3.4 Molecular docking between the multi-epitope subunit vaccine with TLR2 and TLR4

**Figure 4:**
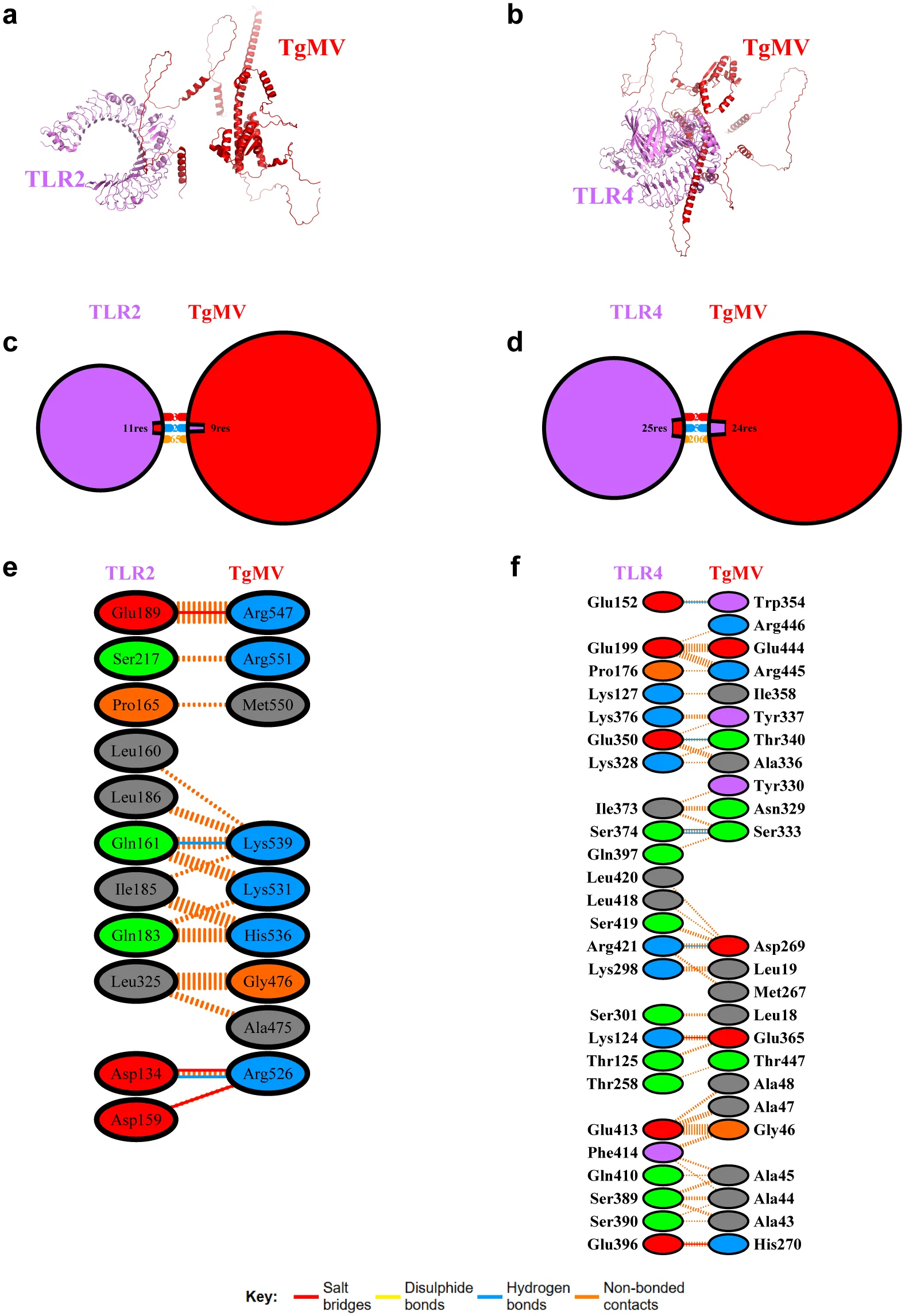
Molecular docking of multi-epitope vaccines with TLR2 and TLR4. **(A, C, E)** Visualization and interaction residues of multi-epitope vaccine and TLR2 complex. **(B, D, F)** Visualization and interaction residues of multi-epitope vaccine and TLR4 complex.

To evaluate the interaction between the multi-epitope vaccine and the natural immune receptors, the HawkDock server was used to perform molecular docking of each with human TLR2 and TLR4. For each docking, the top 100 models were selected based on the docking score and binding free energy. The results showed that the optimal model of the TLR2-vaccine complex had a docking score of -4634.94 and a binding free energy of -32.8 kcal/mol (**Figure 4A**); the optimal model of the TLR4-vaccine complex had a docking score of -4792.41 and a binding free energy of --72.37 kcal/mol (**Figure 4B**). Further, the optimal docking models were visualized and analyzed for interaction through PDBsum. The binding interface of the TLR2-vaccine complex formed a total of 3 salt bridges, 2 hydrogen bonds, and 65 non-covalent contacts (**Figures 4C, E**), with the interface residue numbers of the vaccine and TLR2 being 9 and 11, respectively. The TLR4-vaccine complex formed 2 salt bridges, 5 hydrogen bonds, and 206 non-covalent contacts (**Figures 4D, F**), with the interface residue numbers of the vaccine and TLR4 being 24 and 25, respectively. These results indicate that the designed multi-epitope vaccine has strong interactions with both TLR2 and TLR4.

### 3.5 MD simulation

**Figure 5:**
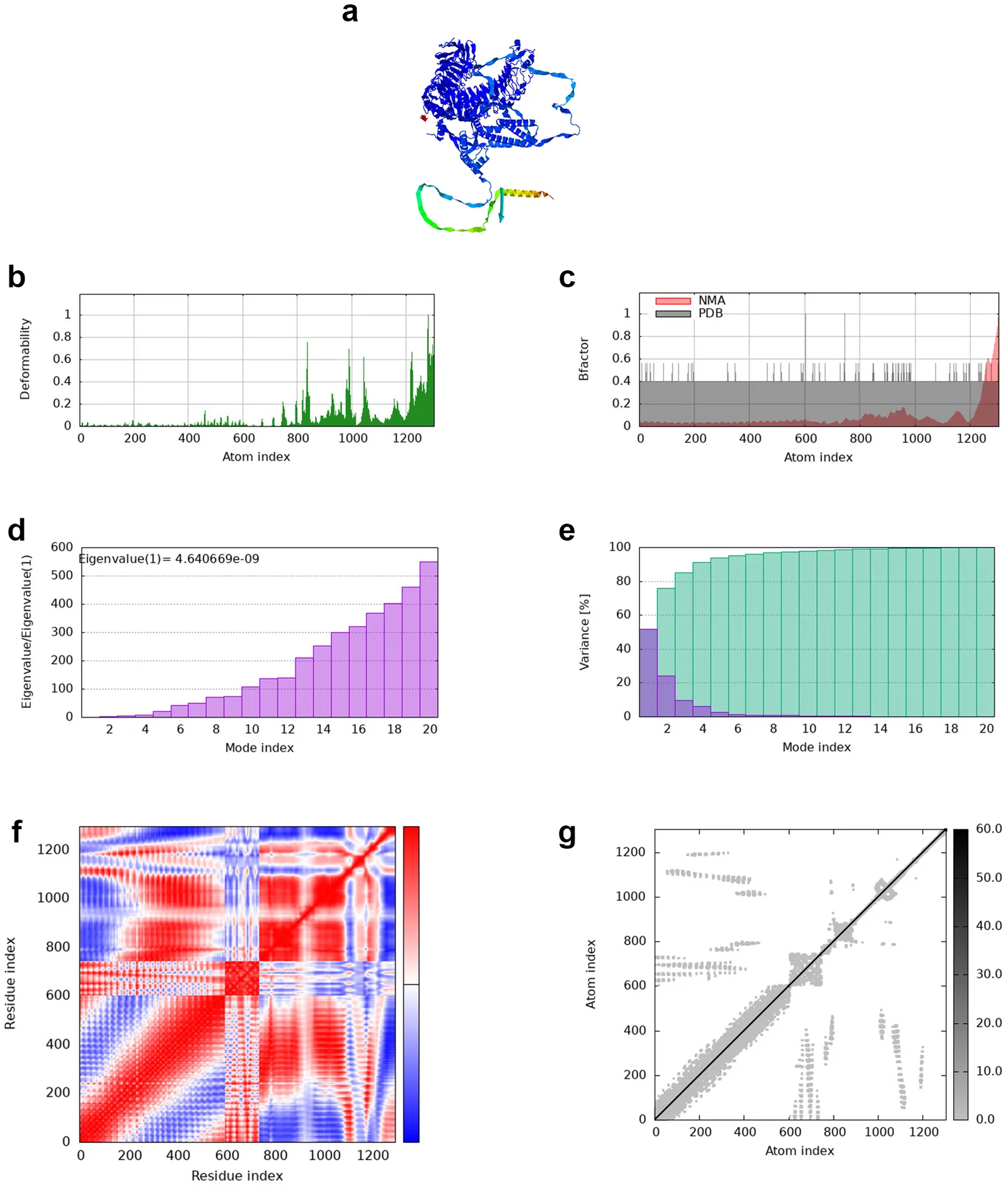
Molecular dynamics simulation of the complex between multi-epitope vaccine and TLR2. **(A)** NMA mobility. **(B)** Deformability plot. **(C)** B-factor plot. **(D)** Eigenvalues plot. **(E)** Variance plot (the individual variance is shown in purple and the cumulative variance is shown in green). **(F)** Covariance plot (red indicates related movements, white indicates unrelated movements, and blue indicates inverse-related movements). **(G)** Elastic network (dark gray represents the harder areas).

**Figure 6:**
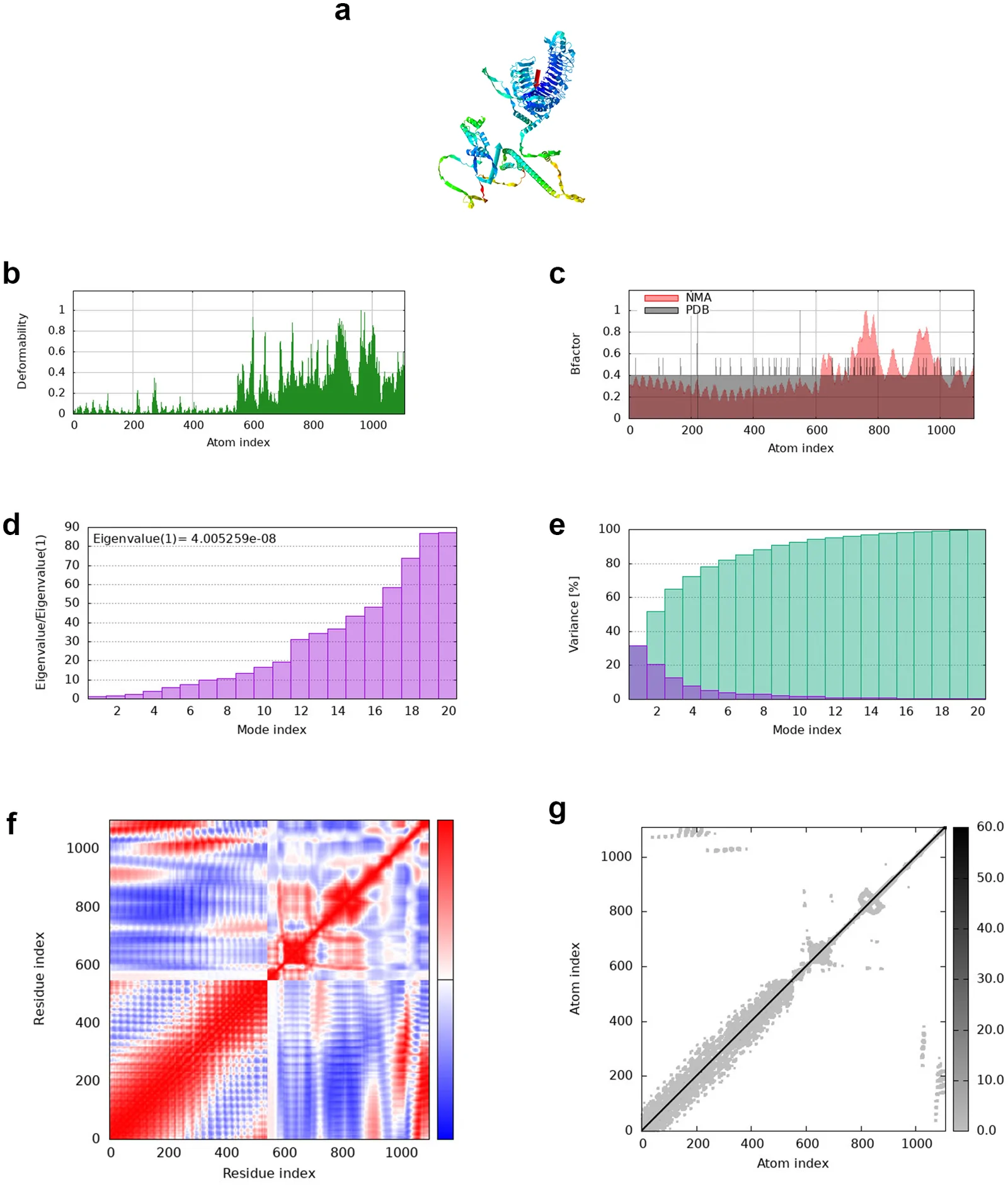
Molecular dynamics simulation of the complex between multi-epitope vaccine and TLR4. **(A)** NMA mobility. **(B)** Deformability plot. **(C)** B-factor plot. **(D)** Eigenvalues plot. **(E)** Variance plot (the individual variance is shown in purple and the cumulative variance is shown in green). **(F)** Covariance plot (red indicates related movements, white indicates unrelated movements, and blue indicates inverse-related movements). **(G)** Elastic network (dark gray represents the harder areas).

To evaluate the stability and dynamic behavior of the vaccine-TLR complexes, molecular dynamics simulations and normal mode analysis were conducted on the optimal docking models using the iMODS network server. The deformation curves of the TLR2-vaccine and TLR4-vaccine complexes were relatively gentle, indicating that both complexes did not undergo significant deformation during the simulation (**Figures 5B**, **6B**). The B-factor curves showed the distribution of root mean square deviation (RMSD) between the normal mode analysis (NMA) and the PDB structure (**Figures 5C**, **6C**). The eigenvalues reflect the energy required for structural deformation, and the lower the eigenvalue, the more easily the α-carbon skeleton deforms. The eigenvalues of the TLR2-vaccine and TLR4-vaccine complexes were 4.005259e-08 and 4.640669e-09, respectively (**Figures 5D**, **6D**), both at a low level, indicating that both complexes have good structural stability. The variance plot shows the contribution of each normal mode to the overall motion, with individual variance and cumulative variance represented in purple and green, respectively (**Figures 5E**, **6E**). The covariance plot is used to characterize the correlation of atomic motions in the complex, with red, white, and blue representing correlated, uncorrelated, and anti-correlated motions, respectively (**Figures 5F**, **6F**). The stiffness analysis based on the elastic network model shows that the hard regions in the complexes are represented by dark gray, while the flexible regions are represented by light dots (**Figures 5G**, **6G**). Based on the above results, both vaccine-TLR complexes exhibit good stability and reasonable dynamic flexibility.

### 3.6 Global population coverage

To assess the potential effectiveness of the designed multi-epitope vaccine across diverse human populations, a population coverage analysis was conducted using the IEDB tool based on the selected CTL and HTL alleles. As shown in Sheet 3 of the **Supplementary Materials**, this candidate vaccine demonstrated an exceptional global coverage of 95.31%. At the regional level, the immunogenic coverage was highest in Europe (98.62%) and North America (95.95%), followed by the West Indies (91.35%) and East Asia (90.62%). Furthermore, substantial coverage was observed in other major regions, including Oceania (87.91%), South Asia (85.49%), and South America (70.25%). These results strongly indicate that this construct possesses broad-spectrum immunogenic applicability and holds great promise as an effective universal preventive vaccine globally.

### 3.7 Immune simulation of the multi-epitope subunit vaccine

**Figure 7:**
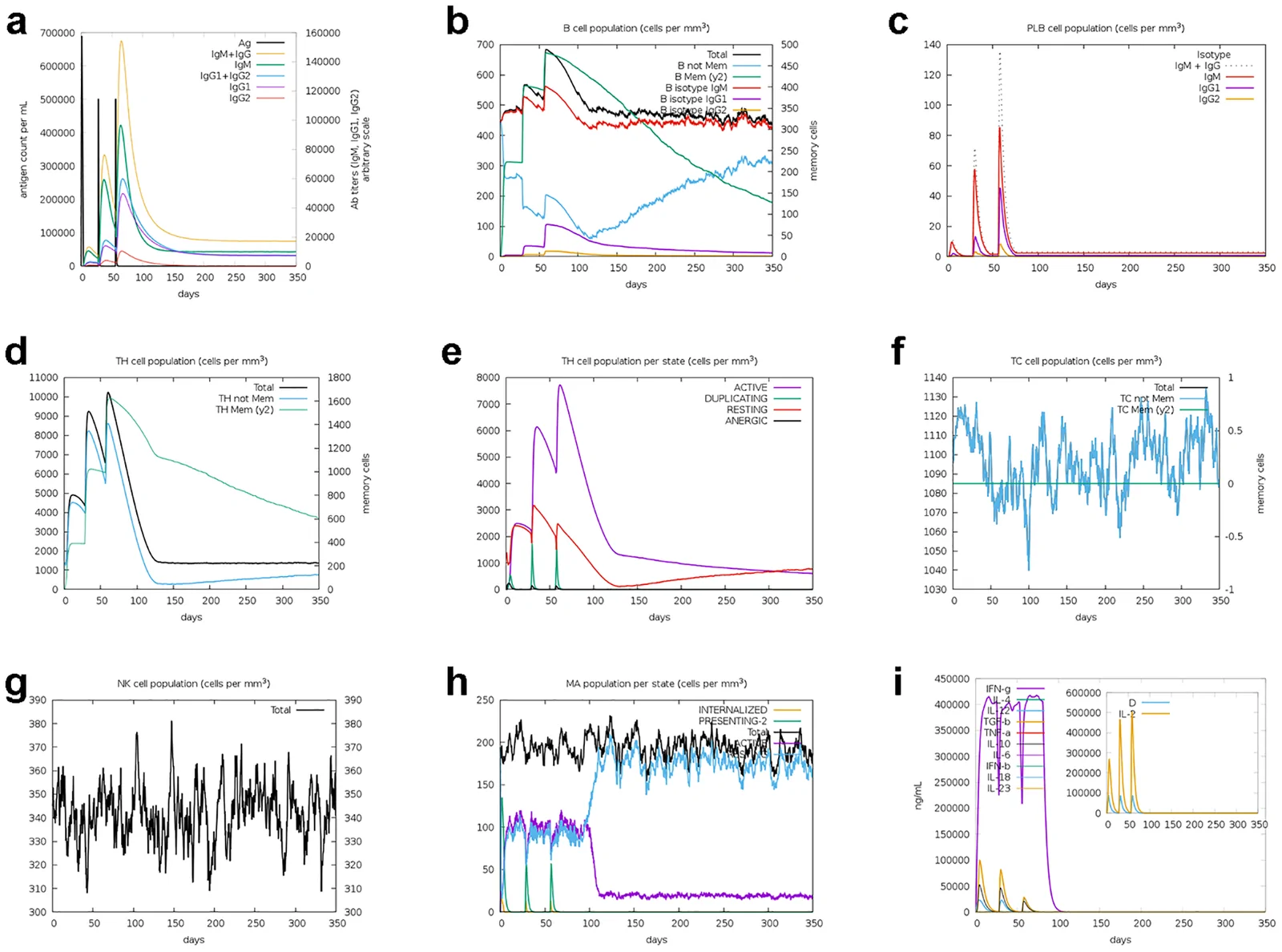
Immune response simulation of multi-epitope vaccine. **(A)** The changes in IgM and IgG antibody titers after three vaccine injections. **(B)** Change in B cell population. **(C)** Changes in the plasma cell population. **(D)** Changes in TH cells. **(E)** Changes in TH cells in different states. **(F)** Changes in Tc cells. **(G)** Changes in NK cells. **(H)** Changes in MA cells. **(I)** The levels of interleukins and cytokines.

The potential of the vaccine to induce adaptive immune responses in the human body was evaluated using the CImmSim network server. The simulation results showed that only low levels of IgM antibodies, IgM^+^ B cells, and a small number of IgG^+^ B cells were detected after the first injection (**Figures 7A, B**). With the second and third injections, the antibody response significantly increased, manifested by an increase in IgM antibody levels, the appearance of various antibody isotypes such as IgG1, IgG1+IgG2, and IgG+IgM, and the generation of a large number of memory B cells (**Figures 7A, B**). In terms of cellular immune responses, the populations of plasma cells, total helper T cells, memory helper T cells, and activated helper T cells all significantly increased after the second and third immunizations (**Figures 7D, E**). Additionally, cytotoxic T lymphocytes (CTL) persisted throughout the immunological simulation, with the maximum count exceeding 1130 cells/mm³ (**Figure 7F**). Natural killer cells and macrophages also showed an activated state (**Figures 7G, H**). Cytokine analysis revealed that the vaccine could induce the production of various cytokines such as IFN-γ, IL-2, and IL-10 (**Figure 7I**). These results indicate that the designed vaccine can effectively activate humoral immunity and cellular immunity, and induce memory immune responses, demonstrating excellent immunogenicity.

### 3.8 Codon optimization and in silico cloning

**Figure 8:**
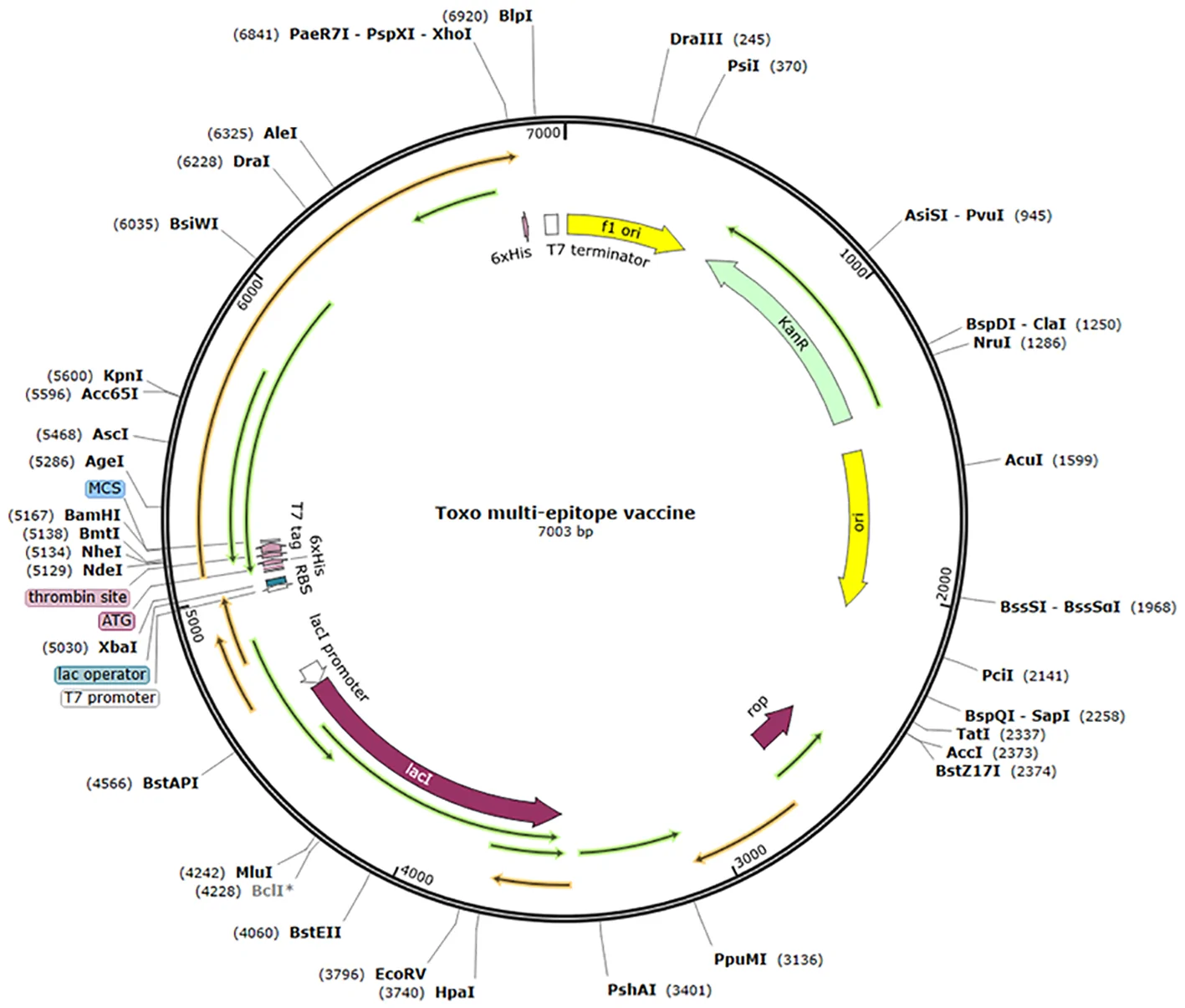
In silico cloning of the multi-epitope vaccine sequence into the pET-28a (+) vector.

To ensure efficient expression of the vaccine in *Escherichia coli* and facilitate production, codon optimization was carried out using the VectorBuilder server. After optimization, the full-length cDNA of the vaccine was 1668 bp, with a codon adaptation index (CAI) of 0.93 and a GC content of 56.43%. All these parameters were within the ideal range, indicating that the vaccine has good expression potential in *Escherichia coli* (K12 strain). Subsequently, the optimized cDNA sequence was cloned into the pET28a(+) expression vector using the SnapGene software (**Figure 8**).

## 4 Discussion

Considering the severe situation of *T. gondii* infection in some regions of the world (such as South America) and the obvious side effects of existing treatment drugs, the development of a human vaccine for the prevention and treatment of this pathogen has become a key task in the field of public health (Jongert et al., 2009). Currently, the main types of *T. gondii* vaccines include attenuated live vaccines, inactivated vaccines, subunit vaccines, and DNA vaccines. Although attenuated live vaccines can induce a relatively strong protective immune response, their potential risk of virulence recovery limits the safety of clinical application. In contrast, inactivated vaccines have higher safety but suffer from the deficiency of insufficient immunogenicity. While subunit vaccines and DNA vaccines have better safety, their protective effects are often relatively limited. In view of these limitations of the existing vaccines, the *T. gondii* multi-epitope vaccine designed based on immunoinformatics methods, due to its advantages such as high safety and strong targeting, is gradually becoming a promising new vaccine strategy with development prospects.

Epitope screening is a crucial step in the design of multi-epitope vaccines. To enhance the immune coverage of the vaccine for different developmental stages of *T. gondii*, this study selected 49 antigens from multiple antigen families such as SAG, GRA, MIC, and ROP for the prediction and screening of CTL, HTL, and B cell epitopes. During the screening process, antigenicity determines whether the epitopes can be recognized by immune cells (Dey et al., 2022), while toxicity and allergenicity are related to human health and safety (Aziz et al., 2022). Given the important roles of IL-4 (Alexander et al., 1998), IL-10 (Gazzinelli et al., 1996), and IFN-γ (Fadaka et al., 2021) in the immune protection against *T. gondii*, this study also predicted the ability of HTL epitopes to induce the production of these cytokines. In the end, a total of 9 CTL epitopes, 6 HTL epitopes, and 11 B cell epitopes were screened. The rational selection of linkers helps to improve the structural stability of the fusion protein (Dong et al., 2020; Fadaka et al., 2021). Based on this, this study selected three short linkers, AAY, GPGPG, and KK, to sequentially link the CTL, HTL, and B cell epitopes obtained from the screening. In addition, although multi-epitope vaccines have higher safety, their immunogenicity is usually weak. Therefore, adjuvants are often needed in practical applications to enhance the immune response (Kwissa et al., 2007). Adjuvants not only enhance the immunogenicity of multi-epitope vaccines but also help induce more durable immune protection (Apostólico et al., 2016). Previous studies have (Lee et al., 2014) shown that 50S ribosomal protein L7/L12 can induce the maturation of dendritic cells (DCs). Mature DCs then activate naive T cells, effectively promoting the polarization of CD4+ and CD8+ T cells and secreting IFN-γ, thereby inducing the cytotoxic response mediated by T cells. Therefore, this study selected it as an adjuvant and used the EAAAK linker to connect with the N-terminus of the vaccine design to increase the immune response.

The final constructed vaccine consists of 556 amino acids and has a molecular weight of 61.13 kDa, which is lower than the reasonable threshold of 110 kDa, indicating its excellent potential for application (Butt et al., 2012). From the perspectives of physicochemical properties and structural characteristics, this vaccine has good antigenicity, stability, and water solubility, and is non-toxic and non-allergenic, meeting the basic requirements of an ideal vaccine candidate. In addition, the solubility prediction and codon optimization results indicate that the expression potential of this vaccine in *Escherichia coli* is high, with CAI values and GC contents both within the ideal range, providing a feasible basis for subsequent experimental verification and large-scale production.

The TLR2 and TLR4 receptors play a certain role in the recognition of *T. gondii* (Debierre-Grockiego et al., 2007). Therefore, this study also explored the docking of the multi-epitope vaccine with these two receptors. Molecular docking technology is an important tool commonly used in drug and vaccine design, capable of predicting the binding ability of vaccine candidate molecules to immune receptors. The docking analysis results show that the binding free energy of the vaccine to these two receptors is -32.8 kcal/mol (for TLR2) and -72.37 kcal/mol (for TLR4), indicating that the vaccine molecule can form stable complexes with the immune receptors. Further analysis indicates that multiple hydrogen bonds and salt bridges have formed between the vaccine molecule and the binding sites of TLR2 and TLR4 receptors, which may enhance the activation effect of the receptors.

The intensity and duration of immune stimulation are among the key indicators for evaluating the efficacy of vaccines. This study utilized the C-ImmSim immunological simulation platform to conduct a systematic assessment of the immune response induced by multi-epitope vaccines. The simulation results showed that after three immunizations, the antibody levels significantly increased, especially the IgG1 subtype antibodies and the number of memory B cells significantly increased. Previous studies have shown that Th1-type immune responses mainly provide protective immunity against *T. gondii*, while Th2-type responses help limit the immune pathological damage caused by infection (Yarovinsky, 2014; Golkar et al., 2007). The simulation results of this study confirmed that the vaccine can stimulate a mixed Th1 and Th2 immune response. In terms of cellular immune responses, the simulation data showed that CTL and HTL cell populations significantly expanded after multiple immunizations, indicating that the vaccine can effectively activate cell-mediated immune responses. Previous studies have confirmed that CD8^+^ T cells and CD4^+^ T cells play indispensable roles in resisting *T. gondii* infection, and the IFN-γ secreted by them can inhibit parasite replication and kill tachyzoites (Hunter and Sibley, 2012; Wang et al., 2017). The immunological simulation of this study also observed a significant increase in IFN-γ levels.

To ensure the practical applicability and translation feasibility of the proposed multi-epitope constructs, choosing an appropriate vaccine delivery strategy is of utmost importance. Compared with other advanced platforms such as DNA or mRNA-based systems, the recombinant protein vaccine platform was selected as our best delivery strategy. Although mRNA and DNA vaccines excel in inducing intracellular antigen processing and show potential in combating various pathogens, they often encounter challenges such as suboptimal *in vivo* delivery efficiency, strict cold chain requirements, or potential genomic integration issues, especially in large-scale veterinary or resource-limited environments (Wang et al., 2024; Pardi and Krammer, 2024). In contrast, the recombinant protein platform offers significant practical advantages. These advantages are strongly supported by our results in silico codon optimization and the stable cloning design within the Escherichia coli pET-28a(+) vector. These advantages include the establishment of a comprehensive high-yield manufacturing scheme, enhanced chemical stability at ambient temperatures, and a significant reduction in production costs (Meena et al., 2022; Wang et al., 2021). Given that toxoplasmosis imposes a significant socio-economic burden in global livestock farming and public health, especially in developing regions where healthcare infrastructure may be limited, an economically efficient, cold chain-independent recombinant subunit protein vaccine represents the most scalable and realistic intervention strategy.

Previous in silico-designed vaccines against T. gondii typically targeted a small number of antigens and often omitted global population coverage assessment (Dodangeh et al., 2019; Hammed-Akanmu et al., 2022). In contrast, the present study screened 49 antigens from four major protein families and integrated CTL, HTL, and B-cell epitopes into a single construct. Furthermore, our design incorporated systematic homology screening against the human proteome and global population coverage analysis, thereby providing a more rigorous evaluation of vaccine safety and population-level applicability. The combined use of dual TLR2/TLR4 docking and AlphaFold3-based structural modeling further enhanced the predictive reliability of the immune activation mechanism, offering advantages over the homology-modeling approaches employed in earlier studies.

Nevertheless, a key limitation of this study is its exclusive reliance on computational and immunoinformatics predictions. Although the structural stability and immunological parameters of the multi-epitope construct have been rigorously evaluated using state-of-the-art algorithms, its actual expression efficiency in bacterial systems, biosafety profile, and *in vivo* protective efficacy await empirical confirmation. Therefore, future work will focus on *in vitro* recombinant protein expression and subsequent *in vivo* challenge studies in animal models to comprehensively assess its capacity to induce robust and protective immune responses.

## 5 Conclusion

In conclusion, the multi-epitope vaccine proposed in this study demonstrates favorable immunogenic, structural and expression-related characteristics in silico. By integrating multiple antigenic components and targeting both innate and adaptive immune pathways, this design may provide a promising framework for vaccine development against human *T. gondii* infections However, its actual immune protection efficacy and application value still need to be verified through subsequent systematic *in vitro* and *in vivo* experiments.

## Data Availability

The original contributions presented in the study are included in the article/**Supplementary Material**. Further inquiries can be directed to the corresponding author.
